# Advanced eco-friendly spectrophotometric analysis for Nebivolol, Valsartan, and related impurity with comprehensive environmental impact assessment

**DOI:** 10.1038/s41598-025-00733-9

**Published:** 2025-05-14

**Authors:** Abdallah M. Hamdy, Magda M. El Henawee, Hisham Hashem, Esraa M. Meselhy, Hany Ibrahim

**Affiliations:** 1 Pharmaceutical Chemistry Department, Faculty of Pharmacy, Egyptian Russian University, Badr City, 11829 Cairo Egypt; 2 Analytical Chemistry Department, Faculty of Pharmacy, Zagazig University, Zagazig, 44519 Egypt

**Keywords:** Double divisor-ratio spectra derivative, Dual wavelength in ratio spectrum, Eco-friendly, H-point derivative ratio, Nebivolol, Valsartan, Analytical chemistry, Green chemistry, Chemistry

## Abstract

At present, one of the main priorities for analysts is creating more sustainable and environmentally friendly methods for pharmaceutical analysis. In this context, three innovative spectrophotometric techniques, using tri-colored (green, blue, and white), were optimized and developed to simultaneously assess two antihypertensive drugs, Nebivolol hydrochloride (NEB) and Valsartan (VAL), in the presence of synthetic precursor impurity of Valsartan (Valsartan Desvaleryl, VAL-D), which is the main acidic degradation product. The methods employed, including the double divisor-ratio spectra derivative spectrophotometric method, the dual-wavelength in ratio spectrum method, and the H-point derivative ratio method, displayed a good linear range of 2.5–70 µg/mL, 10–50 µg/mL, and 10–70 µg/mL for NEB, VAL, and VAL-D, respectively. Assessment of greenness was implemented to the established methods using the analytical Eco-Scale scoring, analytical greenness metric (AGREE), and the green analytical process index (GAPI). The concepts of blueness assessment using the recently introduced Blue Applicability Grade Index (BAGI tool) and whiteness assessment using the Red Green Blue 12 (RGB 12 tool) were also applied. The proposed methods were validated as per ICH guidelines and verified to be accurate. Statistical analysis was performed to ensure the validity of methods concerning published methods.

## Introduction

**Fig. 1:**
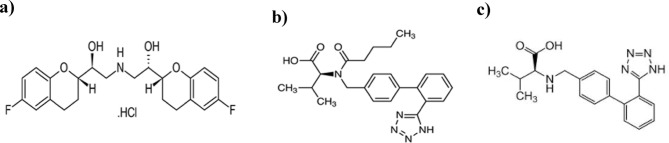
Chemical structure of (**a**) Nebivolol HCl (**b**) Valsartan (**c**) Valsartan Desvaleryl.

One of the main global causes of unexpected death is hypertension^1^. Effective treatment of hypertension nowadays often requires multiple drug combinations^2^. Multidrug single-pill combinations reduce costs and improve patient compliance issues^3^. Nebivolol hydrochloride (NEB)/Valsartan (VAL) is the first combination of an angiotensin receptor blocker and a fixed-dose β-blocker authorized by the FDA^4^. These co-formulated drugs were recently approved in 2016 by the FDA^5^ under the name Byvalson and marketed in India under the name Nebicard V^®^. NEB, represented in Fig. 1a, is (1RS, 1’ RS)-1, 1’- [(2RS, 2’ SR)-bis(6-fluorochroman-2-yl)] -2,2’-iminodiethanol hydrochloride^6^. It is used in the treatment of hypertension because of its vasodilating properties, which seem to be caused by a direct effect on the endothelium, potentially including the release of nitric oxide^6^. VAL, represented in Fig. 1b, is ((2 S)-3-Methyl-2- [pentanoyl [[2’-(1 H-tetrazol-5-yl) biphenyl-4-yl] methyl] amino] butanoic acid^7^. It is used to treat heart failure, control hypertension, and lower cardiovascular death in patients with left ventricular dysfunction following myocardial infarction^6^. VAL-D is represented in Fig. 1c.

Spectrophotometric determination is a straightforward analytical procedure that suits the majority of analytical laboratories’ roles in pharmaceutical analysis without requiring expensive supplies, specialized labor, advanced instruments, or extremely pure solvents. Limited conventional approaches relied on mathematical manipulation and could assess complex mixtures in a less complicated way than the intricate mathematical algorithms stratified in chemometric techniques.

Different analytical techniques were reported for the quantitative determination of their binary mixtures, such as spectrophotometric methods^8–16^, derivative spectrofluorimetric^17^, and chromatographic techniques, which include LC-mass spectrometry^18^, UPLC^19^, HPLC^16,20–27^. However, no publications dealing with green spectrophotometric determination of NEB and VAL in the presence of Valsartan Desvaleryl (VAL-D) have yet been released. VAL-D is a potential impurity found in VAL commercial preparations. It is a VAL synthetic precursor and formed mainly as an acidic degradation product^28–30^. Chromatographic methods (e.g. LC-MS) were reported for the analysis of the process-related impurities as VAL-D in Valsartan tablet dosage form^29^.

In this context, the present work aims to develop three eco-friendly spectrophotometric methods for the assessment of NEB and VAL in the presence of VAL-D. Double divisor-ratio spectra derivative spectrophotometric method (DD-RS-DS), dual-wavelength in ratio spectrum method (DWRS), and H-point derivative ratio method (HDR) were developed, and these are the first spectrophotometric methods dealing with the assessment of NEB and VAL in the existence of the VAL synthetic precursor (VAL-D). In contrast to other approaches like HPLC, HPTLC, and UPLC, these methods are straightforward, economical, time-saving, and don’t need sophisticated equipment. Green analytical methods were used to improve analysts’ health and reduce their negative environmental impact, an increasingly common need in pharmaceutical research. Three distinct measures were used to evaluate the ecological value of the suggested methods: green assessment techniques such as analytical Eco-Scale scoring, analytical greenness metric (AGREE), and green analytical process index (GAPI); blueness assessment using the recently introduced Blue Applicability Grade Index (BAGI tool); and whiteness assessment using the Red Green Blue 12 (RGB 12 tool).

## Experimental

### Instruments

All spectrophotometric measurements were performed using a JASCO dual-beam (Japan) UV-visible spectrophotometer type V-630. The software bundle known as Spectrum Manager II was utilized. The scanning speed may be set up to 1000 nm/min, with a spectral slit width of 2 nm. Quartz cuvettes with a one-cm route length measured the UV (200–400 nm) region’s light absorption.

### Materials and chemicals

NEB and VAL were generously supplied by the National Organization for Drug Control and Research, Cairo, Egypt, with purities of 99.98% and 99.45% w/w, respectively. VAL-D (CAS number 676129-92-3) was obtained from BOC Sciences suppliers in the United States with a purity certification of 98% w/w. Methanol (HPLC grade, VWR, France) was utilized. Nebicard-V^®^ tablets batch No. (CHL1E003) produced by Indian company Torrent Pharmaceutical Ltd. 5 mg of NEB and 80 mg of VAL are present in each tablet.

### Standard solutions

NEB, VAL, and VAL-D standard stock solutions were created by dissolving each drug (10 mg) separately into a 100 mL volumetric flask using 60 mL methanol as a solvent in each flask. The dissolution may be enhanced via sonication; then the volume was adjusted to 100 mL with methanol to obtain 100 µg/mL as the final drug concentration.

## Procedure

### Construction of calibration curves

Three different sets of 10 mL volumetric flasks were quantitatively filled with varying quantities of the standard stock solution (100 µg/mL) of NEB, VAL, and VAL-D. Methanol was added to the flasks to reach the desired final concentration range, which was 2.5–70 µg/mL for NEB, 10–50 µg/mL for VAL, and 10–70 µg/mL for VAL-D. After scanning them at 200–400 nm, the zero-order absorption spectra were saved. Each concentration was measured three times.

#### Double divisor-ratio spectra derivative spectrophotometric method (DD-RS-DS)

In order to assess NEB, the recorded spectra of NEB across the linearity (2.5–70 µg/mL) were divided by the sum of the spectra of (10 µg/mL) VAL and (10 µg/mL) VAL-D as a divisor. Next, the first derivative spectra were achieved. In order to analyze VAL, the recorded spectra of VAL in the linear range (10–50 µg/mL) were divided by the sum of the spectra of (10 µg/mL) NEB and (10 µg/mL) VAL-D as a divisor. Then, the first derivative spectra were gained. For VAL-D determination, the recorded spectra of VAL-D within the linearity range (10–70 µg/mL) were divided by the sum of the spectra of (10 µg/mL) NEB and (10 µg/mL) VAL as a divisor. Then, the first derivative spectra were deduced. Using the double divisor first derivative amplitude and concentration relationship, the calibration graphs for NEB, VAL, and VAL-D were estimated at 297 nm, 262 nm, and 269 nm, respectively. At the selected wavelengths, each drug’s related regression equation was obtained.

#### Dual wavelength in ratio spectrum method (DWRS)

The recorded absorption spectra of the developed solutions of NEB in the linearity equivalent to (2.5–70 µg/mL) were divided by the VAL-D spectrum (10 µg/mL) to assess NEB. The peak amplitudes of the ratio spectrum were measured at the wavelengths 267 and 254 nm, at which VAL exhibits the comparable amplitude. In order to analyze VAL, the produced spectra (10–50 µg/mL) were divided by VAL-D (10 µg/mL) spectrum. The ratio spectra peak amplitudes were generated at 267 and 299 nm, where NEB exhibits a similar amplitude. For VAL-D determination, the saved zero-order absorption spectra of VAL-D across the linearity corresponding to (10–70 µg/mL) were divided by the spectrum (30 µg/mL) of NEB. At 218 and 228 nm, where VAL exhibits a similar amplitude, the peak amplitudes of the ratio spectra were produced. The calibration graphs for each component were made by graphing the amplitude variations at the chosen wavelengths versus the respective concentrations. The regression equation was then calculated.

#### H-point derivative ratio method (HDR)

The recorded absorption spectra for the estimated NEB solutions, ranging from 2.5 to 70 µg/mL, were divided by the VAL-D spectrum (10 µg/mL), resulting in the first derivative of the ratio spectra. The obtained derivative data’s amplitude values were noted at 297 and 304 nm, where VAL absorbance displays identical values and two calibration curves were assembled. For VAL, the documented spectra of VAL across the linearity range (10–50 µg/mL) were divided by the spectrum (10 µg/mL) of VAL-D; subsequently, the first derivative of the ratio spectra was gained. The data at 300 and 292 nm were utilized in calibration curves at which NEB exhibits the same values, and VAL’s regression equation was computed. In order to assess VAL-D, the obtained absorption spectra of VAL-D across a concentration range (10–70 µg/mL) were divided by the spectrum of NEB (30 µg/mL); subsequently, the first derivative of the ratio spectra was obtained. VAL-D’s regression equation was computed after measuring the amplitude values of the resulting derivative data at 245 and 264 nm, where VAL displays the same values.

### Analysis of laboratory-prepared mixtures

In (DD-RS-DS) and (DWRS) methods, various laboratory mixtures were developed from stock solutions of the mentioned analytes. The obtained mixtures involved various amounts of NEB, VAL, and VAL-D in the calibration curve. At (200–400 nm), the evolved series’ spectra were acquired, and the concentration of each component was calculated in each laboratory-prepared mixture as detailed under each technique’s method.

In the HDR method, for assessment of NEB, laboratory-prepared mixtures were estimated by adding aliquots (50, 60, 80, and 100 µg) of NEB to volumetric flasks with a capacity of 10 mL, involving various proportions of NEB, VAL, and VAL-D. Curves for standard additions were created, where the concentrations of the additional NEB samples at both of the chosen wavelengths (297 and 304 nm) were plotted on the X-axis, and the amplitudes of the first derivative of ratio spectra were plotted on the Y-axis.

For VAL, aliquots of (100, 120, 150, and 180 µg VAL) were introduced to volumetric flasks with a capacity of 10 mL, containing accurate proportions of NEB, VAL, and VAL-D. At the two indicated wavelengths (300 and 292 nm), the concentrations of the added VAL samples were indicated on the X-axis, while the amplitudes of the first derivative of the ratio spectra after the addition of VAL standards were displayed on the Y-axis. For VAL-D determination, the development of curves for standard additions involved plotting the concentrations of the introduced VAL-D samples at the two chosen wavelengths (245 and 264 nm) on the X-axis and the amplitudes of the first derivative of the ratio spectra on the Y-axis. For every mixture, two curves were formed at the two selected wavelengths, the point of intersection of which, when extrapolated, represents the H-point and whose abscissa shows each component’s estimated concentration in the mixture.

### Analysis of pharmaceutical preparations

To ascertain the NEB and VAL contents in the medicinal formulation, 10 tablets of Nebicard-V^®^ were weighed, crushed into a fine powder, and combined thoroughly (each tablet has 5 mg of NEB and 80 mg of VAL). After adding powder equal to the mean weight of two tablets, 70 mL of methanol was transferred and the mixture was sonicated for 20 min. After cooling, the volume was completed to the mark with methanol. Subsequently, 0.5 μm Whatman filter paper was used to filter the solution. A 200 mL volumetric flask was filled to the mark with methanol after adding 5 mL of the filtrate. The two medications were measured using aliquots of this filtrate by applying the techniques described in Sect. 3.1.

## Results and discussion

**Fig. 2:**
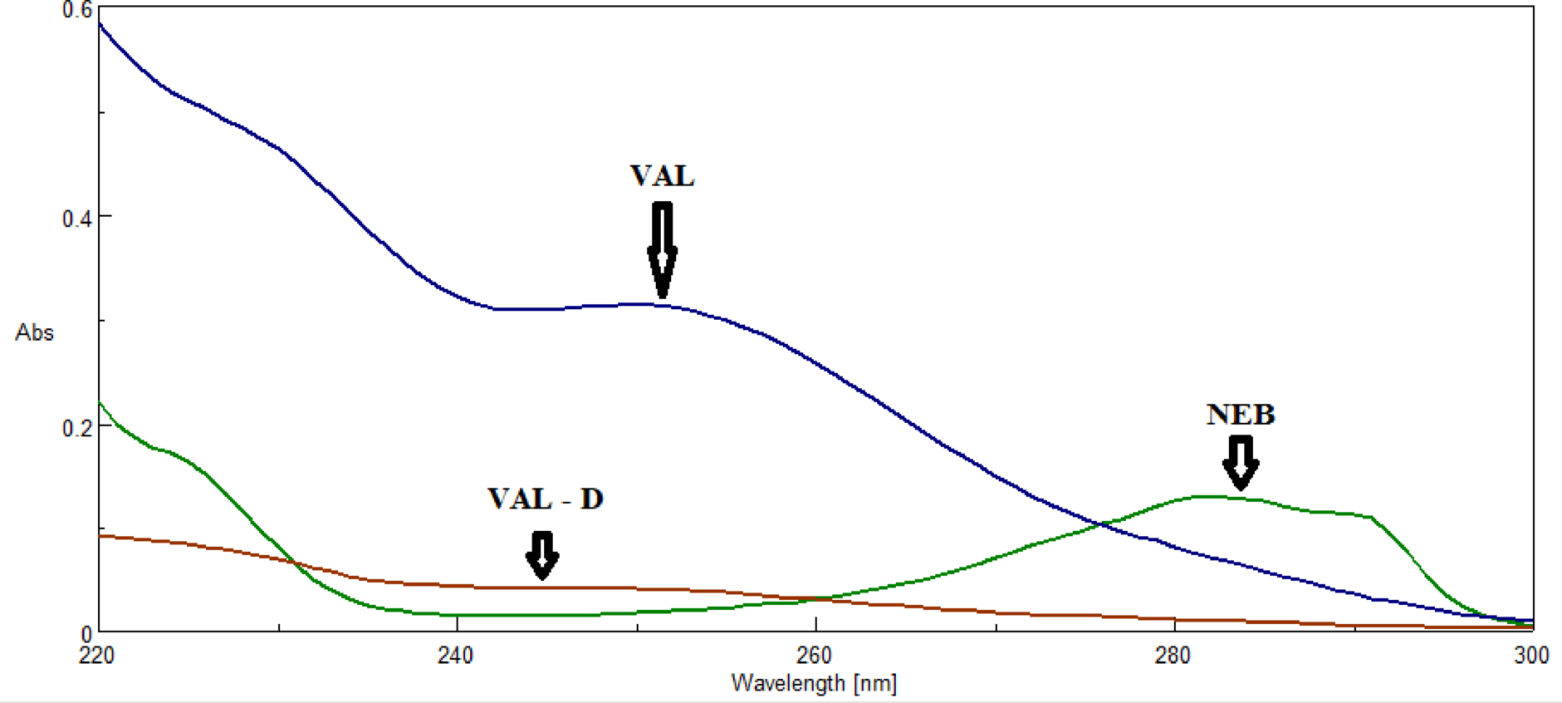
Zero-order absorption spectra (10 µg/mL) of NEB, VAL and VAL-D.

UV spectra of a mixture of NEB and VAL in the presence of VAL-D are severely overlapped, as shown in Fig. 2. Therefore, three economical and environmentally friendly spectrophotometric techniques were created to evaluate NEB, VAL and VAL-D simultaneously in their bulk forms in mixtures generated in the lab, and in their medicinal formulations. VAL-D is a synthetic precursor impurity of valsartan and is a suspected degradation product produced in acidic conditions^28–30^.

### Double divisor-ratio spectra derivative spectrophotometric method (DD-RS-DS)

The use of direct spectrophotometry fails to solve the overlapped of the complicated mixtures. This study developed a method to assess each analyte accurately without interfering. The ternary combination of NEB, VAL, and VAL-D was determined using DD-RS-DS. This method divides each drug’s absorption spectrum by the absorption spectra of a mixture of the other two analytes in the suggested mixture. Each unknown drug concentration in the ternary mixture was determined using its respective drug calibration graph. The calibration curves were realized by charting the amplitudes against their respective drug concentrations at the maximum or minimum wavelengths of each corresponding drug in the ternary mixture^31^.

**Fig. 3:**
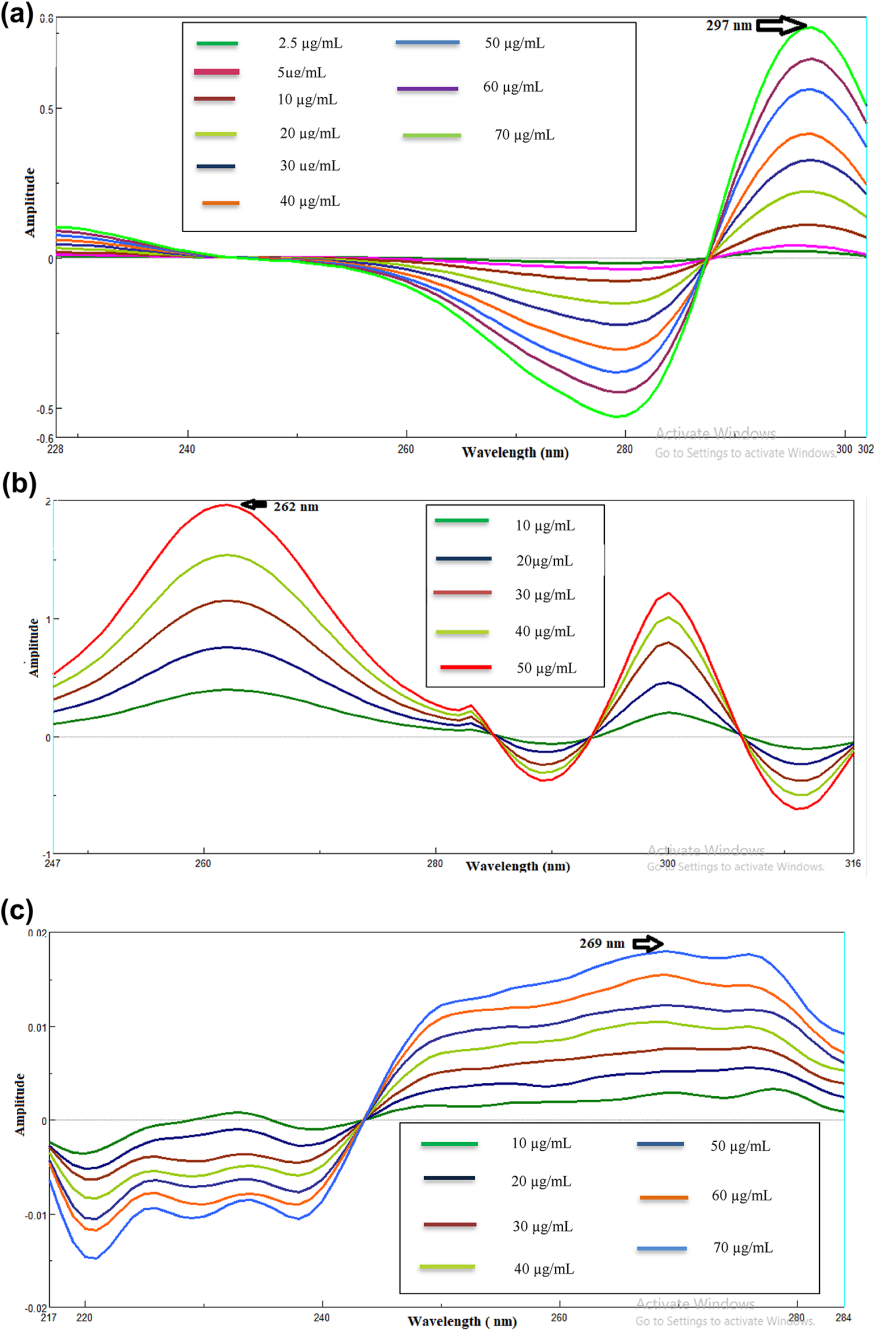
The first derivative of the double divisor-ratio spectra of (**a**) NEB (2.5–70 µg/mL) at 297 nm, (**b**) VAL (10–50 µg/mL) at 262 nm, and (**c**) VAL-D (10–70 µg/ mL) at 269 nm.

To analyze NEB, the obtained spectra across linearity (2.5–70 µg/mL) of NEB were divided by the sum of absorption spectra of (10 µg/mL) VAL and (10 µg/mL) VAL-D as a divisor. Thereafter, double-divisor first-derivative spectra were obtained, represented in Fig. 3a. The relationship between double divisor first derivative amplitude and relevant concentrations was used to create calibration graphs for NEB at 297 nm, and a regression equation was then calculated. For VAL, the obtained spectra across linearity corresponding to (10–50 µg/mL) of VAL was divided by the total absorption spectra of (10 µg/mL) NEB and (10 µg/mL) VAL-D as the divisor. Next, double-divisor first-derivative spectra were attained, represented in Fig. 3b. VAL’s calibration graphs were attained by graphing the double divisor first derivative amplitude on the Y-axis versus their recommended concentrations on the X-axis at the chosen wavelength of 262 nm, after which the equation of regression was calculated. For the VAL-D assessment, the recorded spectra of VAL-D across linearity (10–70 µg/mL) were divided by the sum of the spectra of (10 µg/mL) NEB and (10 µg/mL) VAL as the divisor; afterward, double-divisor first derivative spectra were gained, represented in Fig. 3c. The graphs of calibration for VAL-D were developed to show the relationship between double divisor first derivative amplitude at 269 nm against their respective concentrations, and then the resulting equations of three analytes for regression are shown.

Equations for linear regression were:

$$\begin{gathered} {\mathbf{PA}} = {\text{ }}0.0{\text{111}}{\mathbf{C}} - {\text{ }}0.00{\text{45}}~~~~{\mathbf{r}} = {\text{ }}0.{\text{9999}}~~~{\text{at 297 nm}}~~~{\text{To NEB}} \hfill \\ {\mathbf{PA}} = {\text{ }}0.0{\text{388}}{\mathbf{C}} - {\text{ }}0.00{\text{91}}~~~~{\mathbf{r}} = {\text{ }}0.{\text{9999}}~~~{\text{at 262 nm}}~~~{\text{To VAL}} \hfill \\ {\mathbf{PA}} = {\text{ }}0.000{\text{3}}{\mathbf{C}} + {\text{ }}0.000{\text{3}}~~~~{\mathbf{r}} = {\text{ }}0.{\text{9999}}~~~{\text{at 269 nm}}~~~{\text{To VAL}} - {\text{ D}} \hfill \\ \end{gathered}$$

Where **(PA)** is signified for amplitude of peak, **(r)** represents the coefficient of correlation, and **(C)** is signified for drug concentration measured in (µg/mL).

### Dual wavelength in ratio spectrum method (DWRS)

The ratio difference and dual-wavelength approaches are combined in the dual-wavelength in ratio spectrum methodology to determine ternary mixes. Dual wavelength theory was used to eliminate one interfering component, while the difference in the ratio spectra eliminated the second interfering component, the divisor. In contrast to the ratio difference method, this approach lets us select any wavelength on the spectrum; nevertheless, the wavelength pair we choose needs to have the same amplitude for the ratio spectrum’s first interfering component^32^.

The dual wavelength in the ratio spectrum approach eliminates the interference caused by two components to assess the last in a tripartite combination. It is important to test with various divisor concentrations in order to choose the best divisor that achieves optimal sensitivity. The most suitable divisor of VAL-D (10 µg/mL) provided the best results when it was used to assess NEB and VAL. NEB (30 µg/mL) was used as a divisor to determine VAL-D.

**Fig. 4:**
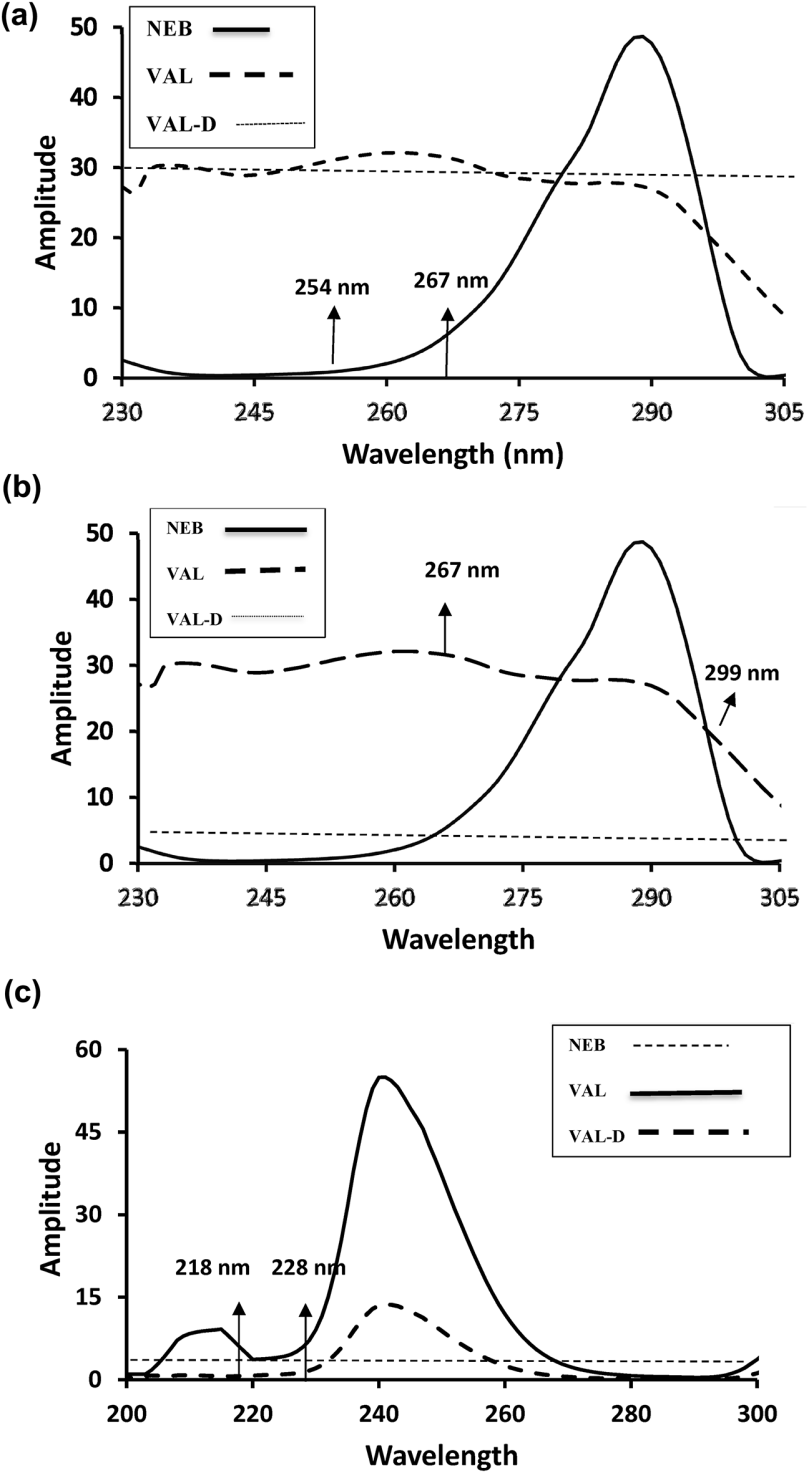
(**a**) Ratio spectra of NEB (———), VAL (– – –) and VAL-D (··········) divided by VAL-D as divisor, illustrating the chosen wavelengths for NEB determination. (**b**) Ratio spectra of NEB (———), VAL (– – –) and VAL-D (··········) divided by VAL-D as divisor, displaying the chosen wavelengths for VAL determination. (**c**) Ratio spectra of NEB (··········), VAL (———) and VAL-D (– – –) divided by NEB as divisor, displaying the wavelengths selected for VAL-D analysis.

The ratio spectra were obtained at two different wavelengths, 267 and 254 nm, to determine the NEB. At 267 and 299 nm, VAL’s ratio spectra’s peak amplitudes were determined. At 218 and 228 nm, the ratio spectra’s peak amplitudes were identified for VAL-D. Using calibration plots, the variations in the amplitudes at the chosen wavelengths were plotted against the associated drug concentrations illustrated in Fig. 4a-c.

The equations for linear regression were:

$$\begin{gathered} \Delta {\mathbf{PD}} = {\text{ }}0.{\text{1799}}{\mathbf{C}} + {\text{ }}0.{\text{1361}}~~~~{\mathbf{r}} = {\text{ }}0.{\text{9999}}~~{\text{To NEB}} \hfill \\ \Delta {\mathbf{PD}} = {\text{ }}0.{\text{3}}0{\text{55}}{\mathbf{C}} + {\text{ 2}}.00{\text{44}}~~~~{\mathbf{r}} = {\text{ }}0.{\text{9995}}~~~{\text{To VAL}} \hfill \\ \Delta {\mathbf{PD}} = {\text{ }}0.0{\text{119}}{\mathbf{C}} + {\text{ }}0.0{\text{298}}~~~~{\mathbf{r}} = {\text{ }}0.{\text{9994}}~~~{\text{To VAL}} - {\text{ D}} \hfill \\ \end{gathered}$$

Where **(∆PD)** is signified to amplitude difference, **(r)** represents the coefficient of correlation, and **(C)** is signified to drug concentration measured in µg/mL.

### H-point derivative ratio method (HDR)

Binary mixtures were assessed using conventional H-point standard additions approach^12^. The H-point approach was used for the absorption spectrum of zero-order binary mixtures, but this approach has poor robustness.

The corresponding study presented a modification to the H-point standard additions approach that improved the method’s ability to resolve spectral overlaps and enabled the method’s study of tripartite combinations. This change is constructed on one of the mixture’s components is canceled by application of the derivative ratio approach. The H-point standard additions approach canceled the second constitute, so it was possible to estimate the third analyte precisely.

For ternary combinations, overlapped spectral data were resolved using the H-point standard additions approach on the first derivative of the ratio spectrum. H-point derivative ratio approach (HDR) combines H-point standard additions with the derivative ratio method to assess the ternary mixture^32^.

For assessment of NEB, NEB absorption, spectra were divided by the VAL-D spectrum (10 µg/mL) to obtain the first derivative of the ratio spectra. At 297 and 304 nm, where VAL exhibits the same values, the obtained derivative data’s amplitude values were noted, and a pair of calibration plots were assembled. To assess VAL, spectrums of the resulting VAL solutions were divided by (10 µg/mL) of VAL-D spectra, and the ratio spectrum’s first derivative was gained. Then, a pair of calibration graphs were created using the data at 300 and 292 nm, where NEB displays similar values. To assess VAL-D, the obtained absorption spectra of VAL-D were divided by (30 µg/mL) of NEB spectra, and the ratio spectrum’s first derivative was gained. Next, a pair of calibration plots were created using the data at 300 and 292 nm, where NEB exhibits the same values.

Using the H-point standard additions principle, the concentration of every analyte in the mixture was determined by adding portions of the constituent to the tripartite combinations. To analyze NEB, laboratory-prepared mixtures were estimated where accurate proportions of NEB (50, 60, 80, and 100 µg) were introduced to volumetric flasks measuring 10 mL that contained a series of the three components. Laboratory-prepared mixtures for VAL were obtained by adding various series of each constituent to 10 mL volumetric flasks containing aliquots (100, 120, 150, and 180 µg) of VAL. For VAL-D, aliquots containing 100, 200, 300, and 400 µg of VAL-D were precisely placed in volumetric flasks measuring 10 mL, including various series of the three components.

Standard addition curves were constructed after the component to be identified was added in incremental amounts. The X-axis showed the appropriate concentrations of the added standard at the two chosen wavelengths, while the Y-axis showed the amplitudes of the first derivative of the ratio spectra of each solution. Two straight lines with distinct intercepts and slopes were produced by plotting the amplitudes versus the additional standard concentrations.

**Fig. 5:**
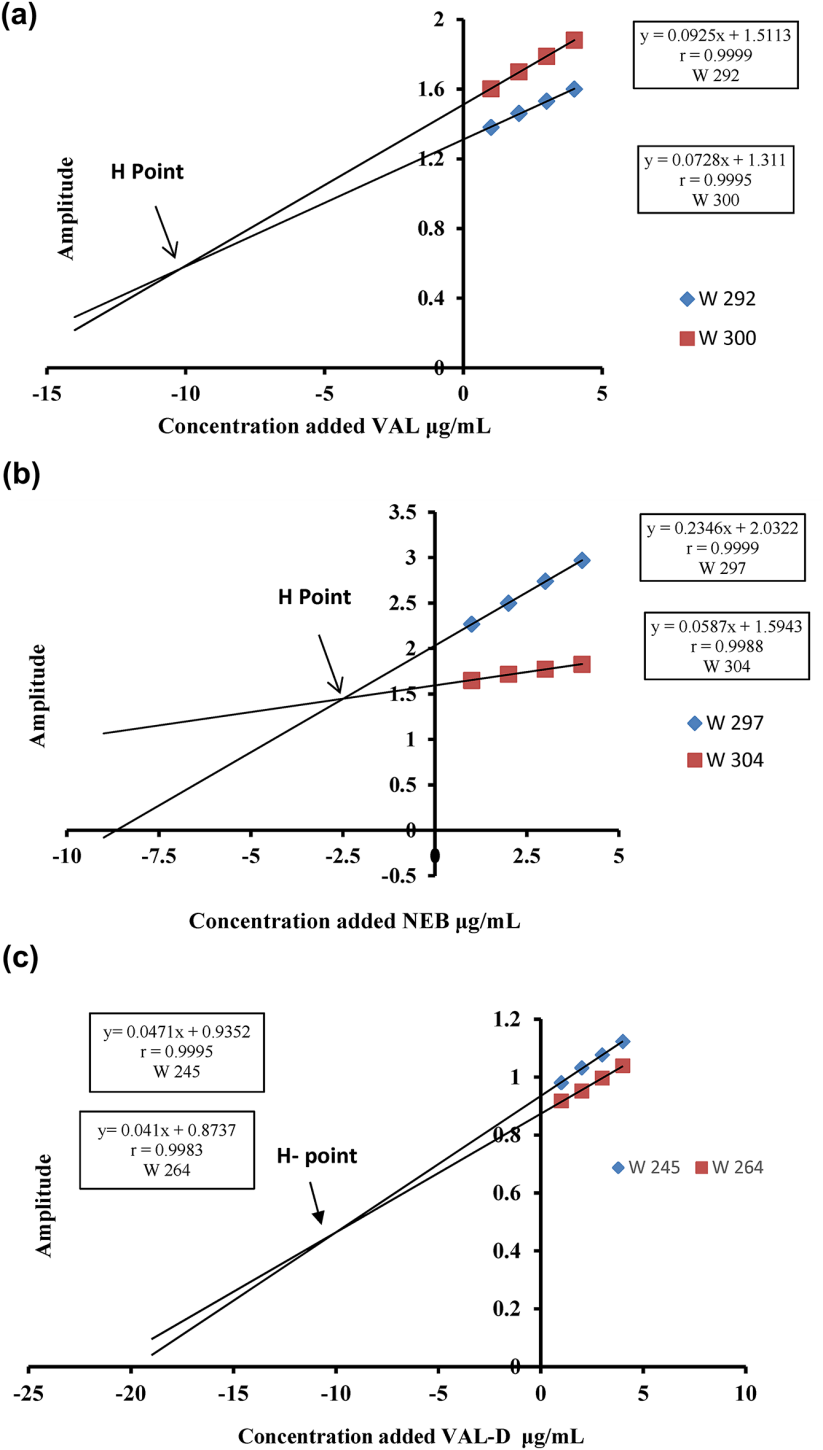
(**a**) Graph of a laboratory-prepared combination using the H-point derivative ratio technique against the appropriate additional concentrations of standard NEB at 304 and 297 nm. (**b**) Graph of a laboratory-prepared combination using the H-point derivative ratio technique against the appropriate additional concentrations of standard VAL at 292 and 300 nm. (**c**) Graph of a laboratory-prepared combination using the H-point derivative ratio technique versus the appropriate additional concentrations of standard VAL-D at 245 and 264 nm.

The concept of H-point standard additions will result in straight lines at different wavelengths intersecting at the H-point, since one component’s value remains constant at the two designated wavelengths. The abscissa showed the concentrations of NEB, VAL, and VAL-D, respectively Figure 5a-c.

## Method validation

The estimation of validation parameters was based on the guidelines of ICH^33^.

### Linearity and range

The linearity of the suggested spectrophotometric techniques was assessed by evaluating various concentrations of VAL-D, VAL, and NEB across linearity ranges (10–70 µg/mL, 10–50 µg/mL, and 2.5–70 µg/mL, respectively) for the double divisor in the ratio spectra derivative spectrophotometric method, the dual wavelength in the ratio spectrum method, and the H-point derivative ratio method. Therefore, the solutions’ absorption spectra were examined at 200–400 nm and then stored. Table 1 shows various regression parameters of the proposed techniques. For detection of impurity, the pharmaceutical dosage form enrichment for impurity was done using a concentration from impurity range.

**Table 1:** Data analysis using the aforementioned spectrophotometric techniques for evaluating NEB, VAL, and VAL-D.

Parameters	Double divisor ratio spectra derivative spectrophotometric method			Dual wavelength in ratio spectra method			H-point derivative ratio method					
	NEB	VAL	VAL-D	NEB	VAL	VAL-D	NEB		VAL		VAL-D	
Range (µg/mL)	2.5–70	10–50	10–70	2.5–70	10–50	10–70	2.5–70		10–50		10–70	
Wavelength selected (nm)	297	262	269	∆P (254–267)	∆P (267–299)	∆P (218–228)	297	304	300	292	245	264
Correlation Coefficient (r)	0.9999	0.9999	0.9999	0.9999	0.9995	0.9994	0.9999	0.9999	0.9997	0.9997	0.9997	0.9997
Slope	0.0111	0.0388	0.0003	0.1799	0.3055	0.0119	0.1249	0.0283	0.0319	0.0188	0.0052	0.0042
Intercept	-0.0045	-0.0091	0.0003	0.1361	2.0044	0.0298	0.0383	0.0382	0.0579	0.0573	0.0012	0.0012
LOD (µg/mL)	0.78	1.08	1.65	0.75	1.94	2.28	0.72	0.73	1.65	1.40	1.38	1.27
LOQ (µg/mL)	2.38	3.27	5.01	2.28	5.87	6.90	2.19	2.23	5.00	4.26	4.20	3.85
Specificity ^**a**^ (Mean ± SD)	100.07 ± 1.19	99.74 ± 1.16	99.92 ± 1.31	101.27 ± 0.71	100.81 ± 1.41	99.62 ± 1.11	100.76 ± 1.85		100.21 ± 1.17		100.26 ± 1.34	
Accuracy ^**b**^ (Mean ± SD)	100.10 ± 0.25	100.27 ± 0.50	99.52 ± 1.29	99.77 ± 1.84	99.41 ± 1.26	98.32 ± 0.30	99.90 ± 0.84		98.94 ± 1.06		100.08 ± 0.82	
Intermediate precision ^c^	1.63	1.15	1.57	1.53	1.21	1.08	1.43		0.88		0.88	
Repeatability Precision ^d^	1.86	1.04	1.88	1.34	1.27	1.53	1.60		0.82		1.23	

^a^Specificity (average % recoveries ± standard deviation) of the mixtures’ recovery percentage statistics prepared in the laboratory.

^b^Accuracy (average % recoveries ± standard deviation) investigated with a minimum of nine measurements over a minimum of three different concentration values within the given range.

^c^The intraday (*n* = 3) three concentrations’ RSD repeated three times throughout the day.

^d^The interday (*n* = 3) three concentrations’ RSD repeated three times over three consecutive days.

### Limit of detection (LOD) and limit of quantification (LOQ)

LOD and LOQ were utilized to evaluate the sensitivity of suggested techniques. The subsequent formulas were used to calculate LOD and LOQ, relying on utilizing the intercept’s standard deviation (σ) and slope of the calibration plot (S) Table 1.

$$\begin{gathered} {\text{LOD }} = {\text{ }}\left( {{\text{3}}.{\text{3 }} \times {\text{ }}\sigma } \right){\text{ }}/{\text{ S}} \hfill \\ {\text{LOQ }} = {\text{ }}\left( {{\text{1}}0{\text{ }} \times {\text{ }}\sigma } \right){\text{ }}/{\text{ S}} \hfill \\ \end{gathered}$$

### Accuracy

To evaluate the accuracy of the proposed approaches, three concentrations of the three analytes were measured in triplicate (20, 40, and 60 µg/mL for VAL-D and NEB and 10, 30, and 50 µg/mL for VAL). Table 1 displays the average recovery percentages for each drug that was determined.

### Precision

Three separate concentrations of the suggested medications, within the linear range, were analyzed by three replication analyses of three pure analyte samples (20, 40, and 60 µg/mL) for NEB as well as VAL-D and (10, 30, and 50 µg/mL) for VAL, on one day and three days later to determine the intra-day and inter-day precision of the suggested procedures; the results were illustrated in Table 1.

### Specificity

The aforementioned techniques’ specificity was estimated by assessing several laboratory-prepared combinations involving various amounts of NEB, VAL, and VAL-D inside the calibration graph. The mixtures of (NEB: VAL: VAL-D) in the following concentrations: (20:20:20), (20:20:10), (30:30:10), (10:10:30), (10:10:20), and (2.5:40:10) were prepared. The recoveries and standard deviations obtained from these mixtures were within acceptable limits Table 1.

## Application to pharmaceutical preparation

The aforementioned methodologies effectively estimated NEB and VAL in their co-formulated dosage form (Nebicard V^®^) tablets. By applying the standard addition approach to test the validity of the proposed procedures, no influence from excipients was observed Table 2.

**Table 2:** Assessment of pharmaceutical Preparation and utilization of standard addition approach.

Technique	Analyte	Proposed Method *R*%^*^ ± SD	Standard addition *R*%^**^ ± SD
Double divisor-ratio spectra in ratio spectra derivative spectrophotometric method (DD-RS-DS)	NEB	99.32 ± 0.79	99.92 ± 1.58
	VAL	100.11 ± 0.67	100.15 ± 1.72
Dual wavelength in ratio spectrum method (DWRS)	NEB	99.73 ± 0.44	99.94 ± 1.62
	VAL	99.95 ± 0.31	100.09 ± 1.22
H-point derivative ratio method (HDR)	NEB	100.01 ± 0.89	99.27 ± 0.47
	VAL	100.35 ± 0.47	99.81 ± 1.35

^*^Average of three determinations (The label of a Nebicard V^®^ tablet states that each tablet includes 80 mg of VAL and 5 mg of NEB).

^**^ mean of six assessments through three concentration levels.

For detection of the impurity, the pharmaceutical dosage form enrichment to impurity was done by the addition of a concentration of 20 µg/mL of impurity to the pharmaceutical formulation. For the impurity recovery calculations, the added 20 µg/mL concentration recovery is subtracted from the total recovery obtained after pharmaceutical formulation enrichment.

## Assessment of greenness

Green analytical chemistry aims to reduce the harm that chemical analysis causes to the environment. It encourages the adoption of ecologically friendly reagents, methods, and technology. Researchers’ focus is on using the fundamentals of green analytical chemistry to develop more Eco-friendly analytical techniques^34–37^.

### Analytical Eco-scale scoring

The analytical Eco-scale (penalty points) scoring system is a unique quasi-quantitative methodology used to estimate the greenness of the offered procedures. It is based on the fact that the ideal green analysis has a score of 100, which serves as the principle of an Eco-Scale analysis^38^. If the analytical technique deviates from the ideal green analysis, penalty points (PPs) are given for every parameter (number of reagents, risks, energy, and waste). Then, the following formula should be used to calculate the Eco-Scale scoring, considering the total penalty points for the entire procedure.

$${\text{Analytical Eco}} - {\text{Scale }} = {\text{ 1}}00{\text{ }} - {\text{ total }}\left( {{\text{PPs}}} \right)$$

Calculation results are evaluated on a scale, with > 75 implying excellent green analysis, 50–75 implying acceptable green analysis, and < 50 implying inadequate green analysis. The suggested spectrophotometric methods had penalty points (PPs) of 12, as shown in Table 3. The results of the Eco-scale reflect excellent and acceptable greenness for the spectrophotometric method, which is equal to 88, as demonstrated in Table 3.

**Table 3:** The outcomes of the evaluation of the suggested approaches’ greenness.

Analytical eco-scale score		Analytical greenness metric (AGREE)	Green analytical procedure index (GAPI)
Item	Penalty points		
Reagents			
Methanol	6		
Instrument			
Spectrophotometer			
Energy (˃ 0.1kWh per sample)	0		
Occupational hazard	0		
Waste (1–10 mL, no treatment)	6		
Total PPs	12		
Analytical Eco-scale score ^a^	Σ 88		

Analytical eco-scale score = 100 (ideal score).

> 75 (great green analysis).

50–75 (acceptable green analysis).

< 50 (inadequate green analysis).

### Analytical greenness metric (AGREE)

AGREE—Analytical greenness metric, wherein the input variables correspond to the 12 fundamentals of Green Analytical Chemistry (GAC). Each variable is converted into a standardized scale ranging from 0 to 1, as outlined in the twelve fundamentals of GAC. The final evaluation result is the sum of the evaluation outcomes for each method. The resulting outcome exhibits a circular pictogram, wherein the central region displays the sum of the score and its associated color representation. The achievement of the technique in every principle is displayed using a straightforward red-yellow-green color scale, as shown in Table 3.

As methanol is the solvent used during the overall analysis, so the criterion 11 model is not met according to the AGREE model (It is suggested to consider any material as toxic if it is defined as toxic via inhalation, ingestion, dermal contact, or toxicity to aquatic life.). However, this point could be improved by using greener solvents, while the criterion 7 could be enhanced by decreasing the solvent amount used (miniaturization) and consequently decreasing the waste generation. Also, the criteria 1 & 5 could be improved via direct/online and automation of analytical procedures, which results in lower occupational exposure, especially toward vapors of solvents, and the risk of accidents is also reduced.

### Green analytical process index (GAPI)

GAPI is a new technique for greenness assessment that reflects ecological impact through five pentagrams to symbolize different degrees of environmental impact of each stage of the analytical methodology: mild, moderate, and severe, as illustrated in Table 3.

GAPI model displays a color-coded pentagram representing various phases during the overall analytical method e.g. sample treatment and preparation, reagents/solvents, waste generation and instrumentation. The score can be improved towards an ideal value of 1 via direct analysis without any sample preparation steps. The solvent used should be replaced with greener one alongside reducing solvent consumption and recycling the used solvent.

## Assessment of blueness “BAGI tool” and whiteness “RGB 12 tool”

The BAGI is a useful instrument that links to assessing analytical methods’ sustainability and provides readers with information about method simplicity. This tool’s free software produces two important indicators. While a method scoring more than 60 is regarded as very relevant, the first indicator has a middle score. The second metric evaluates the extent of the blue coverage in the asteroid pictogram; a darker blue suggests greater applicability, while a white background shows that the criteria have not been met. According to Table 4, our methods demonstrated their outstanding practical applicability, high production capacities, automation potential, and exceptionally low operating costs by achieving an impressive BAGI total score of 85^39^.

**Table 4:** The outcomes of the evaluation of the suggested approaches “whiteness and blueness”.

Method	
BAGI tool (blueness assessment)	
RGB 12 tool (whiteness assessment)	

We developed the RGB12 algorithm to analyze the whiteness profile of our proposed methods. This algorithm is a helpful tool for quantitatively evaluating the overall sustainability level of analytical procedures. The RGB12 method has 12 distinct algorithms categorized into three groups: blue, green, and red. Every set of algorithms addresses significant issues pertaining to sustainability. The G1–G4 green category focuses on important aspects like poisoning, wasted energy, additional reagents, and potential impacts on people, animals, and altered species. The red group assesses validation criteria, including the method’s applicability, LOD and LOQ, accuracy, and precision (R1–R4). The blue category (B1–B4) evaluates aspects like affordability, ease of use, practicality issues, and financial needs. Utilizing the RGB 12 algorithm, an extensive evaluation is carried out to confirm the solutions’ exceptional efficacy and demonstrate how they align with sustainability principles. This assessment focuses on the suggested methods’ improved performance, dependability, and economic viability, making them a desirable alternative to several analytical instruments Table 4^40,41^.

### Statistical analysis

The recommended approaches and the stated method were compared statistically. This is accomplished by calculating t- and f-values that are less than the theoretical values, which show that the proposed approaches and the published technique^25^ did not differ significantly, as illustrated in Table 5. An additional statistical comparison was achieved utilizing the one-way ANOVA test at a 5% significance level by using the recovery % gained after the application of the three investigated approaches on the pharmaceutical dosage form. The results proved that no discernible difference existed between the groups, as illustrated in Table 6. The test demonstrated that the suggested approaches and the reference method^25^ did not differ significantly (*P* > 0.05).

**Table 5:** A statistical analysis of the published and suggested approaches for assessing the pure form of VAL and NEB.

Item	(DD-RS-DS)		(DWRS)		(HDR)		Published method^25^	
	NEB	VAL	NEB	VAL	NEB	VAL	NEB	VAL
Mean	99.32	100.11	99.73	99.95	100.01	100.35	**99.68**	**99.85**
SD	0.79	0.67	0.44	0.31	0.89	0.47	**0.55**	**0.49**
RSD	0.80	0.67	0.44	0.31	0.89	0.47	**0.55**	**0.49**
Variance	0.62	0.45	0.19	0.096	0.79	0.22	**0.30**	**0.24**
n	6	6	6	6	6	6	**6**	**6**
Student^’^s *t*-test	0.92 (2.23)	0.77 (2.23)	0.17 (2.23)	0.42 (2.23)	0.77 (2.23)	1.80 (2.23)	–	–
*F*- test	2.06 (5.05)	1.87 (5.05)	1.56 (5.05)	2.50 (5.05)	2.62 (5.05)	1.09 (5.05)	–	–

The values enclosed in parenthesis corresponded to the appropriate t and F tabulated values at (*P* = 0.05).

**Table 6:** Results of One–way ANOVA for comparison of the proposed methods and the reported method^25^.

	Source of Variation	Sum of Squares	Degree of freedom	Mean Square	F ^a^	Sig.
NEB	Between Methods ^c^	0.723	3	0.241	0.534 (5.05^b^)	0.672
	Within Methods	3.610	8	0.451		
	Total	4.334	11			
VAL	Between Methods ^c^	0.421	3	0.140	0.491 (5.05^b^)	0.698
	Within Methods	2.286	8	0.286		
	Total	2.707	11			

^a^F is the ratio of mean square to error mean square.

^b^The tabulated value of F.

^c^Between the reported method^25^ and the three corresponding methods (DD-RS-DS), (DWRS), and (HDR).

## Conclusion

Simultaneous determination of a ternary mixture without a prior separation step was realized via three cost-effective spectrophotometric methods. Eco-friendly analytical methods are used in the current work to improve analysts’ health and reduce their negative environmental impact. In the pharmaceutical industry, stability testing is crucial to ascertain how a medication product’s quality varies over time due to environmental and laboratory circumstances. This work aims to provide three accurate, reliable, and straightforward spectrophotometric techniques for assessing our mixture in their laboratory mixtures and its marketed formulation tablet without the need for laborious sample processing or interference from additives in pharmaceutical formulations. NEB and VAL in their ternary mixes, as well as in medicinal preparations, can all be accurately determined using the procedures that have been outlined. The techniques were authorized as per the ICH guidelines.

## Data Availability

The datasets used and/or analyzed during the current study are available from the corresponding author on reasonable request.
